# Palmitic Acid Lipotoxicity in Microglia Cells Is Ameliorated by Unsaturated Fatty Acids

**DOI:** 10.3390/ijms22169093

**Published:** 2021-08-23

**Authors:** C.J. Urso, Heping Zhou

**Affiliations:** Department of Biological Sciences, Seton Hall University, South Orange, NJ 07079, USA; cj.urso@student.shu.edu

**Keywords:** fatty acid uptake, lipid droplets, lipoprotection, lipotoxicity, neutral lipid accumulation, palmitic acid, unsaturated fatty acids

## Abstract

Obesity and metabolic syndrome are associated with cognitive decline and dementia. Palmitic acid (PA) is increased in the cerebrospinal fluid of obese patients with cognitive impairment. This study was therefore designed to examine fatty acid (FA) lipotoxicity in BV2 microglia cells. We found that PA induced time- and dose-dependent decrease in cell viability and increase in cell death without affecting the cell cycle profile and that PA lipotoxicity did not depend on cell surface free fatty acid receptors but rather on FA uptake. Treatment with sulfosuccinimidyl oleate (SSO), an irreversible inhibitor of fatty acid translocase CD36, significantly inhibited FA uptake in BSA- and PA-treated cells and blocked PA-induced decrease in cell viability. Inhibition of ER stress or treatment with N-acetylcysteine was not able to rescue PA lipotoxicity. Our study also showed that unsaturated fatty acids (UFAs), such as linoleic acid (LA), oleic acid (OA), α-linolenic acid (ALA), and docosahexaenoic acid (DHA), were not lipotoxic but instead protected microglia against PA-induced decrease in cell viability. Co-treatment of PA with LA, OA, and DHA significantly inhibited FA uptake in PA-treated cells. All UFAs tested induced the incorporation of FAs into and the amount of neutral lipids, while PA did not significantly affect the amount of neutral lipids compared with BSA control.

## 1. Introduction

Free fatty acids (FFAs) are central to many critical cellular functions serving as cellular energy substrates, participating in cell signaling as receptor ligands and second messengers, and contributing to the structural integrity of cells as integrants of cellular membranes [1]. When in excess, long-chain saturated fatty acids (SFAs) may lead to lipotoxicity, while unsaturated fatty acids (UFAs) exhibit low lipotoxicity or are lipoprotective in many non-adipose cells [2]. FFA uptake in the brains of obese patients with metabolic syndrome is elevated compared to healthy subjects [3]. The level of palmitic acid (PA), a principal FFA constituent of human plasma [4] and a key SFA in membrane phospholipids [5], is elevated in the cerebrospinal fluid of overweight and obese subjects and this elevation is associated with decreased cognitive performance [6]. Indeed, SFA-induced functional abnormalities in neuronal and glial cells have been suggested to contribute to cognitive decline and neurodegenerative conditions [7], while UFA-containing fish oil supplementation decreases apoptosis in hippocampus and cerebrum of rodents [8,9].

The mechanisms by which elevated SFAs evoke pathological states are not clearly defined. There is evidence suggesting that fatty acids may act as extracellular signaling ligands by binding to a family of cell surface G protein-coupled receptors called free fatty acid receptors (FFARs) [10,11,12,13]. Among them, FFAR1 and FFAR4 are activated by long-chain SFAs and UFAs [14]. Different FFAR-dependent functions have been reported in various cells. α-linolenic acid (ALA) acts on FFAR1 to induce differentiation of anti-inflammatory M2 macrophages [15]. Linolenic acid (LA) induces the release of glucagon-like peptide 1 from human epithelial NCI-H716 cells via FFAR4 [16]. Pharmacological inhibition of FFAR1 with DC260126 protects MIN6 β cells from PA-induced endoplasmic reticulum (ER) stress and apoptosis [17]. FFAR4 activation has been reported to protect against lipotoxicity-induced pancreatic β cell dysfunction [18]. FFAR1 and FFAR4 have been reported to act in concert in the hypothalamus to reduce energy efficiency and regulate obesity-associated inflammation [19]. FFAR1 and FFAR4 are both detected in BV2 microglia, with FFAR4 expressed at a higher level than FFAR1 [19]. Eicosapentaenoic acid (EPA) reportedly inhibits the NLR family pyrin domain containing 3 (NLRP3) inflammasome activation through FFAR1 and FFAR4 in BV2 microglia cells [20].

Conversely, the differential effects of lipotoxic SFAs and lipoprotective UFAs may depend on their uptake or arise intracellularly. Among the FA transporters reported to facilitate FA uptake, CD36 is highly expressed in primary mouse microglia cells and BV2 microglia [21]. After being transported into cells, FFAs are converted into fatty acyl-CoAs, which may then be catabolized to generate energy or anabolized to produce a range of molecules, including second messengers, hormones, and diacylglycerols (DAGs). DAGs may subsequently be converted to triacylglycerols (TAGs) and incorporated into lipid droplets (LDs) [22]. LDs are cytoplasmic organelles that play important roles in various cellular functions, including storage of neutral lipids and energy metabolism [23]. UFAs have been reported to enhance the synthesis of neutral lipids and the formation of LDs [24]. Co-treatment with arachidonic acid (AA) reduces PA-induced lipotoxicity in C2C12 myotubes, and this AA-induced reduction in PA lipotoxicity is associated with increased formation of LDs [24]. Furthermore, PA has been reported to induce oxidative stress and endoplasmic reticulum (ER) stress in a variety of cells such as rat cortical cells [25], bovine endometrial cells [26], H9C2 cardiomyocytes [27], L6 skeletal muscle cells [27,28], and pancreatic β cells [29], which may be alleviated by co-treatment with UFAs [25], N-acetylcysteine (NAC), an antioxidant [26,27,28], or 4-phenylbutyric acid (4-PBA), an inhibitor of ER stress [27].

Microglia are resident immune cells that constantly monitor the brain environment and maintain the homeostasis of the brain by orchestrating neuroinflammatory responses to various pathophysiological conditions [30]. They are densely populated in gray matter areas, including hippocampus, basal ganglia, and substantia nigra [31]. Studies have suggested that fatty acids may affect the physiology and function of microglia [30]. Microglia dystrophy has been linked with β-amyloid deposits due to futile microglial reaction to insoluble amyloid deposits [32]. While several studies have examined how different FFAs may affect the immune activation of microglia [33,34], the lipotoxic effects of elevated levels of FFAs on microglia have not been investigated. Considering that FFAs may affect different cells in distinct ways, this study was therefore designed to examine PA-induced lipotoxicity and the potential role of FFAR1/FFAR4 and FA uptake in PA lipotoxicity in microglia cells, and how UFAs may protect microglia against PA lipotoxicity. Our studies may provide useful insights into the mechanisms underlying obesogenic cognitive decline and dementia.

## 2. Results

### 2.1. Concentration- and Time-Dependent Inhibition of Microglia Viability by PA

**Figure 1:**
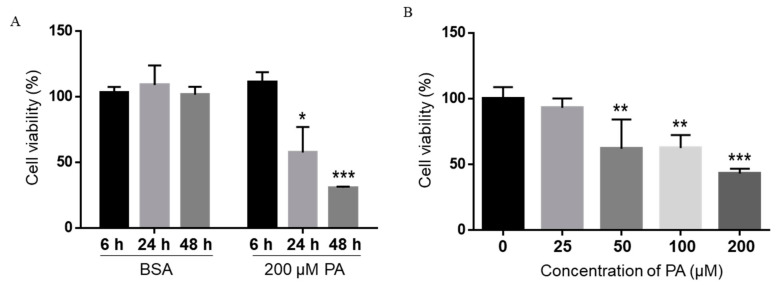
Time- (**A**) and concentration-dependent (**B**) effects of PA on the viability of BV2 cells. BV2 cells were treated with 200 µM PA for different periods of time (**A**) or different concentrations of PA for 24 h (**B**) in serum-free media, and their viability was assessed by MTT assay. The values presented were representative of three independent experiments (mean ± SD). Statistical analysis was performed using two-way ANOVA followed by Tukey’s multiple comparison test. * *p* < 0.05 vs. time-equivalent BSA control; ** *p* < 0.01 vs. time-equivalent BSA control; *** *p* < 0.001 vs. time-equivalent BSA control.

Treatment with 200 µM PA and 200 µM OA for 24 h has been reported to increase non-mitochondrial respiratory rates and induce distinct metabolic profiling in BV2 microglia cells [35]. Therefore, we first treated BV2 microglia cells with 200 µM PA or BSA control for 6, 24, or 48 h in serum-free media, and cell viability was assessed by the 3-(4,5-dimethylthiazol-2-yl)-2,5-diphenyltetrazolium bromide (MTT) assay. Microglia cell viability was not decreased by PA at 6 h post-treatment, but significantly decreased at 24 h and continued to decrease at 48 h following treatment with 200 µM PA (Figure 1A). We also treated microglia cells with different concentrations of PA for 24 h, and their viability was measured by MTT assay. The viability of BV2 cells was significantly decreased following treatment with 50 µM PA for 24 h and continued to decrease as the concentration of PA increased to 100 and 200 µM (Figure 1B).

### 2.2. PA Induced Microglia Cell Death

**Figure 2:**
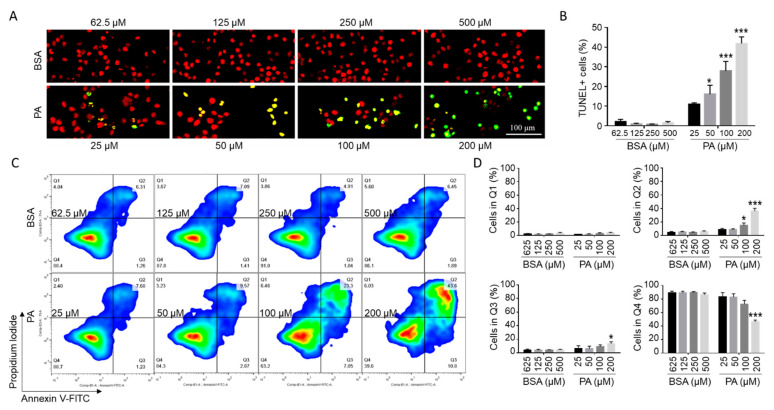
PA-induced microglial cell death. (**A**,**B**) Detection of cell death by TUNEL assay (green) with nuclei stained with PI (red) following treatment with different concentrations of PA and BSA control for 24 h with representative confocal images presented in A and quantitated data presented in B (mean ± SE). (**C**,**D**) Detection of cell death by flow cytometry after staining the cells with FITC-conjugated Annexin V and PI following treatment with different concentrations of PA and BSA control for 24 h in serum-free media with representative flow cytometry contour plots shown in C and quantitated data for each quadrant shown in D (mean ± SE). Top left quadrant (Q1) represented Annexin V^−^/PI^+^ necrotic cells. Top right quadrant (Q2) represented Annexin V^+^/PI^+^ late apoptotic and/or necrotic cells. Bottom right quadrant (Q3) represented Annexin V^+^/PI^−^ early apoptotic cells. Lower left quadrant (Q4) represented Annexin V^−^/PI^−^ live cells. The values presented were representative of three independent experiments (mean ± SE). Statistical analysis was performed using two-way ANOVA followed by Tukey’s multiple comparison test. * *p* < 0.05 vs. corresponding BSA control; *** *p* < 0.001 vs. corresponding BSA control.

To examine whether the PA-induced decrease in cell viability was associated with an increase in apoptosis in microglia, BV2 cells were subjected to terminal deoxynucleotidyl transferase dUTP nick end labeling (TUNEL) assay with fluorescein-12-dUTP following treatment with different concentrations of PA or BSA control for 24 h. TUNEL^+^ staining was barely detectable (0–4%) in cells treated with different concentrations of BSA (Figure 2A,B). A 24 h treatment with 25 µM PA increased TUNEL^+^ cells to 10.0–12.3%. As the concentration of PA increased, the percentage of TUNEL^+^ cells continued to increase. A total of 18.4–33.5% cells were TUNEL^+^ following 24 h treatment with 100 µM PA, and 37.1–48.3% cells were TUNEL^+^ following 24 h treatment with 200 µM PA (Figure 2A,B).

To confirm that PA induced apoptosis, BV2 cells were treated with different concentrations of PA or BSA control for 24 h, stained by Annexin V-FITC and PI, and then analyzed by flow cytometry. As indicated in Figure 2C,D, for cells treated with different concentrations of BSA control, 79–91% cells were Annexin V^−^/PI^−^, 1.9–5.6% cells were Annexin V^−^/PI^+^, 3.6–8.7% cells were Annexin V^+^/PI^−^, and 1.9–6.2% cells were Annexin V^+^/PI^+^. A 24 h treatment with 100 µM PA increased the percent of Annexin V^+^/PI^+^ and Annexin V^+^/PI^−^ cells to 10.7–23.3% and 7.1–16.0%, respectively. A 24 h treatment with 200 µM PA further increased the percent of Annexin V^+^/PI^+^ and Annexin V^+^/PI^−^ cells to 25.8–43.6% and 12.8–18%, respectively (Figure 2C,D). These data confirmed that PA induced apoptosis in microglia cells. The percent of Annexin V^+^/PI^+^ cells was much higher than the percent of Annexin V^−^/PI^+^ cells. It is possible that Annexin V^+^/PI^+^ cells may include necrotic cells.

### 2.3. FFAR Antagonists Did Not Ameliorate PA Lipotoxicity

**Figure 3:**
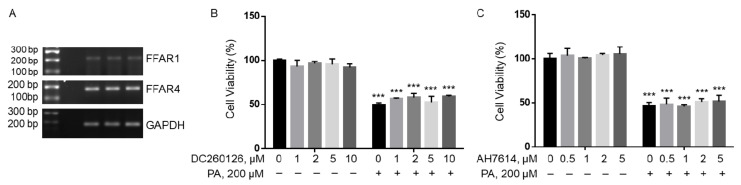
Viability of BV2 cells pre-treated with DC260126 or AH7614 followed by treatment with BSA or PA. (**A**) mRNA expression of FFAR1 and FFAR4 as detected by semi-quantitative RT-PCR with glyceraldehyde 3-phosphate dehydrogenase (GAPDH), a house-keeping gene, as the control. (**B**,**C**) BV2 cells were pre-treated with different concentrations of DC260126 (**B**), a GPR40 antagonist, or AH7614 (**C**), a GPR120 antagonist, for 1 h followed by treatment with 200 µM PA or equivalent volume of BSA in serum-free media for 24 h, and their viability was assessed by MTT assay. The values presented were representative of three independent experiments with triplicate measurements (mean ± SD). Statistical analysis was performed using two-way ANOVA followed by Tukey’s multiple comparison test. *** *p* < 0.001 vs. BSA control.

Long-chain fatty acids have been found to act as extracellular ligands for FFAR1 and FFAR4 [36,37,38]. We found that both FFAR1 and FFAR4 were expressed in BV2 microglia cells, but FFAR1 mRNA was significantly lower than FFAR4 mRNA (Figure 3A). BV2 cells were then pre-treated with different concentrations of DC260126, an antagonist of FFAR1, or AH7614, an antagonist of FFAR4, followed by treatment with 200 µM PA or BSA for 24 h, and their viability was analyzed by MTT assay. Incubation with DC260126 did not significantly affect BV2 viability at all concentrations tested in BSA-treated cells, nor did it ameliorate PA-induced decrease in cell viability (Figure 3B). Similarly, AH7614 did not significantly affect BV2 viability at all concentrations tested, nor did it attenuate PA-induced decrease in cell viability (Figure 3C). These data suggest that PA lipotoxicity may not be mediated by FFAR1 or FFAR4 in BV2.

### 2.4. Inhibition of Fatty Acid Uptake Ameliorated PA Lipotoxicity

**Figure 4:**
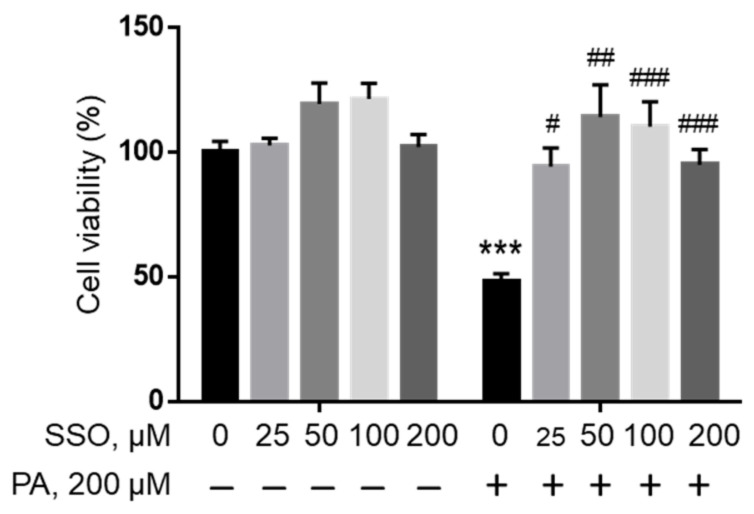
Viability of BV2 cells pre-treated with SSO followed by treatment with BSA or PA. BV2 cells were pre-treated with different concentrations of SSO, a CD36 inhibitor, for 1 h followed by treatment with 200 µM PA or equivalent volume of BSA in serum-free media for 24 h, and their viability was assessed by MTT assay. The values presented were representative of three independent experiments (mean ± SD). Statistical analysis was performed using two-way ANOVA followed by Tukey’s multiple comparison test. *** *p* < 0.001 vs. BSA control; # *p* < 0.05 vs. 200 µM PA; ## *p* < 0.01 vs. 200 µM PA; ### *p* < 0.001 vs. 200 µM PA.

In order to examine whether PA needs to be transported into cells to induce lipotoxicity, sulfosuccinimidyl oleate (SSO), a widely used irreversible inhibitor of fatty acid translocase/CD36 [39], was used to block fatty acid uptake in microglia cells. BV2 cells were pre-treated with different concentrations of SSO followed by treatment with 200 µM PA or BSA control for 24 h, and cell viability was examined by MTT assay. As shown in Figure 4, SSO started to rescue PA lipotoxicity at a concentration as low as 25 µM. Higher concentrations of SSO continued to protect microglia cells from PA lipotoxicity.

**Figure 5:**
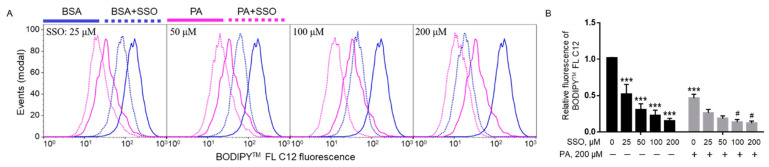
Uptake of BODIPY^TM^ FL C12 in BV2 cells pre-treated with SSO for 1 h followed by treatment with PA or BSA for 6 h. Cells were pre-treated with different concentrations of SSO for 1 h and then treated with 200 µM PA or BSA for 6 h. At the end of treatment, cells were incubated with BODIPY^TM^ FL C12 for 30 min, and fatty acid uptake was measured by flow cytometry with representative histograms shown in (**A**) and quantitated data shown in (**B**). Data presented (mean ± SEM) were representative of three independent experiments. Statistical analysis was performed using two-way ANOVA followed by Tukey’s multiple comparison test. *** *p* < 0.001 vs. BSA control; # *p* < 0.05 vs. 200 µM PA.

In order to confirm that SSO indeed blocked fatty acid uptake, the uptake of 4, 4-difluoro-5, 7-dimethyl-4-bora-3a,4a-diaza-*s*-indacene-3-dodecanoic acid (BODIPY^TM^ FL C12), a fluorescent fatty acid analog, in the presence of SSO in BV2 cells was examined by flow cytometry. BODIPY^TM^ FL C12 was deemed as a suitable fluorescent PA analogue since its chain length is equivalent to PA with the BODIPY^TM^ FL fluorophore accounting for another approximate length of a four-carbon acyl chain while BODIPY^TM^ FL C16 is considered equivalent to arachidic acid (C20:0). Furthermore, metabolic tracing performed in zebrafish has also revealed that the complex lipid product profile of ^3^H-PA most closely resembles that of BODIPY^TM^ FL C12 rather than BODIPY^TM^ FL C16 [40]. Therefore, BODIPY^TM^ FL C12 was deemed as a suitable fluorescent PA analogue to be used in our FA uptake assay. Cells were pre-treated with different concentrations of SSO for 1 h and then treated with 200 µM PA or BSA for 6 h. At the end of treatment, uptake of BODIPY^TM^ FL C12 was analyzed by flow cytometry. A 200 µM PA treatment decreased the uptake of BODIPY^TM^ FL C12 in microglia cells as compared to BSA control. SSO started to significantly decrease BODIPY^TM^ FL C12 uptake at a concentration as low as 25 µM in BSA-treated cells. As the concentration of SSO increased, it continued to decrease BODIPY^TM^ FL C12 uptake in BSA-treated cells (Figure 5A,B). SSO treatment also decreased BODIPY^TM^ FL C12 uptake in PA-treated cells at 25 and 50 µM, and this decrease was statiscally significant at 100 and 200 µM (Figure 5A,B).

### 2.5. Unsaturated Fatty Acids Abolished PA-Induced Decrease in Microglia Viability

**Figure 6:**
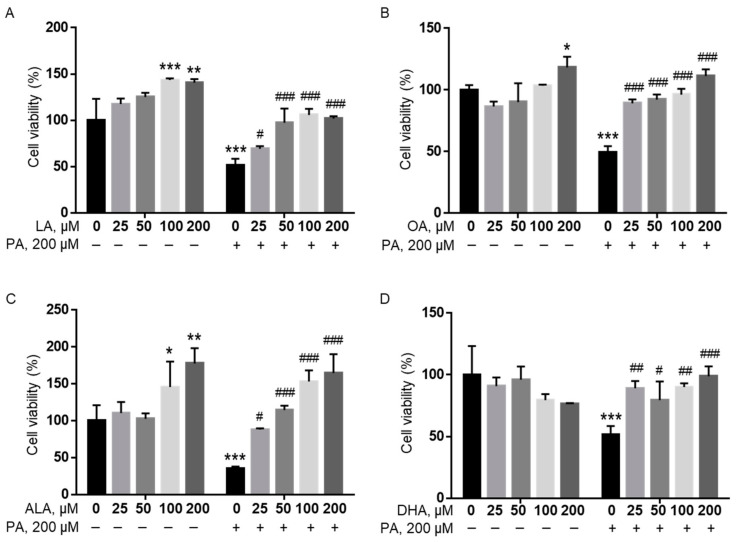
PA-induced decrease in BV2 viability was abolished by LA (**A**), OA (**B**), ALA (**C**), and DHA (**D**). BV2 cells were treated with 0, 25, 50, 100, or 200 µM of LA (**A**), OA (**B**), ALA (**C**), and DHA (**D**) together with 200 µM PA or equivalent volume of BSA control in serum-free media for 24 h, and their viability was assessed by MTT assay. The values presented were representative of three independent experiments (mean ± SD). Statistical analysis was performed using two-way ANOVA followed by Tukey’s multiple comparison test. * *p* < 0.05 vs. BSA control; ** *p* < 0.01 vs. BSA control; *** *p* < 0.001 vs. BSA control; # *p* < 0.05 vs. 200 µM PA; ## *p* < 0.01 vs. 200 µM PA; ### *p* < 0.001 vs. 200 µM PA.

To investigate whether unsaturated fatty acids (UFAs) mitigated the effects of PA on BV2 viability, cells were treated with 25, 50, 100, or 200 μM of linoleic acid (LA), oleic acid (OA), ALA, and docosahexaenoic acid (DHA), together with 200 μM PA or BSA in serum-free media for 24 h, and cell viability was determined by MTT assay. All UFAs examined were found to significantly attenuate the PA-induced decrease in microglia viability starting at concentrations as low as 25 µM regardless of the number and location of their double bonds. At 100 and 200 µM, all UFAs examined completely abolished the PA-induced decrease in BV2 cell viability (Figure 6).

### 2.6. Differential Effects of FFAs on Fatty Acid Uptake in BSA- and PA-Treated Cells

**Figure 7:**
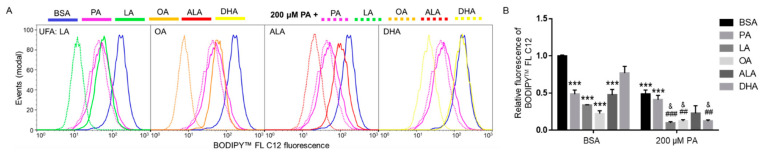
Uptake of BODIPY^TM^ FL C12 in BV2 cells following treatment with BSA or PA together with different FFAs. Cells were treated with BSA or 200 µM PA together with 200 µM of different FFAs in serum-free media for 6 h. At the end of treatment, cells were incubated with BODIPY^TM^ FL C12 for 30 min and FA uptake was measured by flow cytometry with representative histograms shown in (**A**) and quantitated data shown in (**B**). Data presented (mean ± SEM) were representative of three independent experiments. Statistical analysis was performed using two-way ANOVA followed by Tukey’s multiple comparison test. *** *p* < 0.001 vs. BSA control; ## *p* < 0.01 vs. 200 µM PA; ### *p* < 0.001 vs. 200 µM PA; & *p* < 0.05 vs. 400 μM PA.

We next examined whether different FFAs affected the uptake of fatty acids. BV2 cells were treated with 200 µM of different FFAs, including LA, OA, ALA, DHA, and PA with BSA as control for 6 h, and uptake of BODIPY^TM^ FL C12 was examined by flow cytometry. The amount of BODIPY^TM^ FL C12 fluorescence in cells treated with 200 µM PA, LA, OA, or ALA was significantly lower than that in cells treated with BSA, while 200 µM DHA did not significantly decrease BODIPY^TM^ FL C12 uptake as compared to BSA-treated cells. The difference in the amount of BODIPY^TM^ FL C12 uptake in cells treated with 200 µM PA, LA, OA, or ALA did not reach statistical significance (Figure 7).

Since co-treatment with UFAs abolished PA lipotoxicity, we also examined whether co-treatment with additional 200 µM of FFAs affected FA uptake in cells treated with 200 µM PA. As shown in Figure 7, LA, OA, and DHA significantly decreased the amount of BODIPY^TM^ FL C12 uptake in PA-treated cells. Co-supplementation with additional 200 µM ALA also reduced the amount of BODIPY^TM^ FL C12 uptake in PA-treated cells, but the decrease was not statistically significant. Co-supplementation with additional 200 µM PA did not significantly affect BODIPY^TM^ FL C12 uptake compared to cells treated with 200 µM PA, which suggests that 200 µM PA may have saturated the protein transporters for BODIPY^TM^ FL C12. In contrast, additional supplementation with UFAs decreased BODIPY^TM^ FL C12 uptake in PA-treated cells, suggesting that UFAs may affect FA transport via alternative mechanisms instead of direct competition with PA for the FA transporters.

### 2.7. Differential Effects of FFAs on Incorporation of BODIPY^TM^ FL C12 into and Accumulation of Neutral Lipids

**Figure 8:**
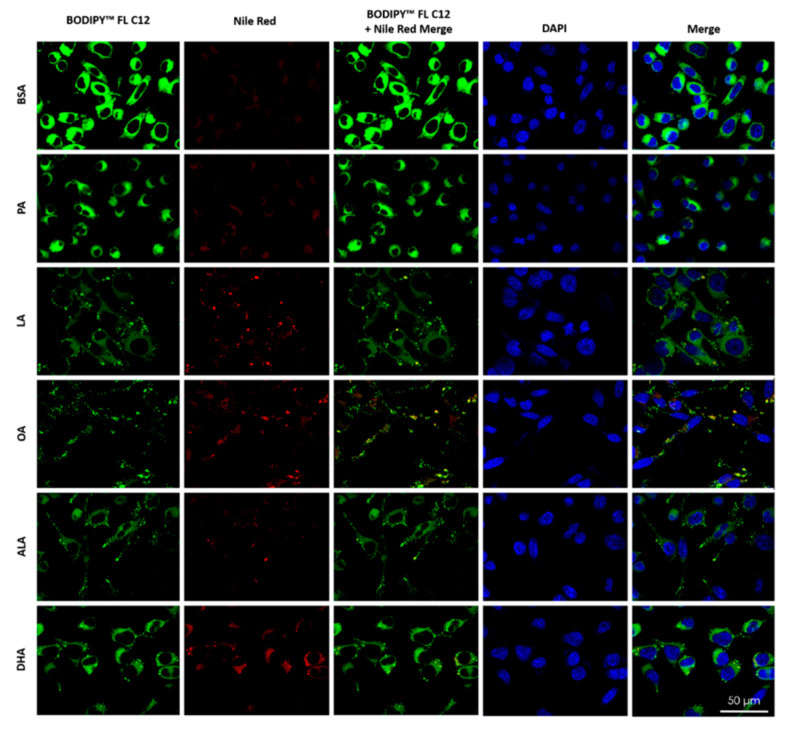
Uptake of BODIPY^TM^ FL C12 and its incorporation into neutral lipids and LDs in BV2 cells. BV2 cells were grown on glass coverslips and treated with 200 µM FFA or BSA control in serum-free media for 6 h. At the end of treatment, cells were incubated with BODIPY^TM^ FL C12 (green) for fatty acid uptake, stained with Nile Red (red) for neutral lipids, and stained with DAPI (blue) for nuclei, washed, mounted, and visualized under confocal microscopy.

To examine the metabolic fate of intracellular BODIPY^TM^ FL C12, cells were treated with 200 µM PA, LA, OA, ALA, DHA, or BSA for 6 h, exposed to BODIPY^TM^ FL C12 for 0.5 h, stained with Nile Red for neutral lipids, and then examined under confocal microscopy. The amount of BODIPY^TM^ FL C12 uptake in BSA-treated cells was higher than that in PA-treated cells, and the amount of BODIPY^TM^ FL C12 uptake was lower in cells treated with LA, OA, or ALA than that in PA-treated cells (Figure 8), which is consistent with our flow cytometry analysis of BODIPY^TM^ FL C12 uptake (Figure 7). The amount of neutral lipid staining by Nile Red in cells treated with 200 µM PA was minimal and comparable to the basal level staining in BSA-treated cells. Treatment with 200 µM LA, OA, ALA, or DHA dramatically enhanced the amount of neutral lipid staining as compared to cells treated with BSA or PA, suggesting that there was increased neutral lipid accumulation in UFA-treated cells. Neutral lipid staining in UFA-treated cells appeared more particulate while that in BSA- and PA-treated cells was more diffuse, suggesting that there was more formation of lipid droplets (LDs) in UFA-treated cells. Moreover, the particulate neutral lipid staining was colocalized with the green fluorescence from BODIPY^TM^ FL C12, suggesting that BODIPY^TM^ FL C12 was incorporated into neutral lipids and LDs in UFA-treated cells (Figure 8).

### 2.8. UFAs Enhanced Neutral Lipid Staining in PA-Treated Cells

**Figure 9:**
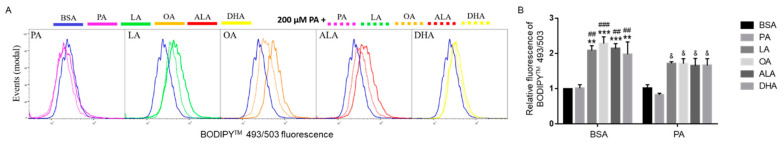
Neutral lipid accumulation in BV2 cells following treatment with BSA or 200 µM PA together with different FFAs. Cells were treated with BSA or 200 µM PA together with 200 µM PA, LA, OA, ALA, or DHA in serum-free media for 6 h. At the end of treatment, cells were incubated with BODIPY^TM^ 493/503 for 30 min followed by flow cytometry analysis of neutral lipid content with representative histograms shown in (**A**) and quantitated data shown in (**B**). Data (mean ± SEM) presented were representative of three independent experiments. Data were analyzed using two-way ANOVA followed by Tukey’s multiple comparison test. ** *p* < 0.01 vs. BSA control; *** *p* < 0.001 vs. BSA control; ## *p* < 0.01 vs. 200 µM PA; ### *p* < 0.001 vs. 200 µM PA; & *p* < 0.05 vs. 400 μM PA.

To examine whether UFAs enhanced neutral lipid accumulation in PA-treated cells as compared to BSA-treated cells, BV2 microglia were incubated with BSA or 200 µM PA together with 200 µM of different FFAs for 6 h, and then the amount of neutral lipids was detected with BODIPY™ 493/503 by flow cytometry. The amount of neutral lipid staining in PA-treated cells was at the basal level comparable to BSA-treated cells. Different UFAs, namely LA, OA, ALA, and DHA, increased the amount of neutral lipids by ~ 100% as detected by BODIPY™ 493/503 (Figure 9), which is consistent with Nile Red staining of neutral lipids in Figure 8. As shown in Figure 9, co-supplementation of 200 µM UFAs, namely LA, OA, ALA, and DHA, significantly enhanced the amount of neutral lipid staining in PA-treated cells while supplementation with an additional 200 µM PA did not affect the amount of neutral lipid staining in PA-treated cells. These data suggest that increased neutral lipid accumulation was not simply due to additional supplementation with any fatty acids. Instead, the increased neutral lipid accumulation was promoted by additional supplementation with UFAs, but not PA.

## 3. Discussion

Lipotoxicity refers to cellular toxicity caused by excess FFAs [41]. PA, the most abundant saturated fatty acid in plasma circulation [42], has been reported to induce apoptotic cell death in many types of cells, including neonatal rat ventricular myocytes [43], β cells [44], skeletal muscle cells [28], liver cells [45,46], podocytes [47], and hypothalamic neurons [48]. While PA may affect the production of inflammatory mediators in microglia [33,34,49], PA lipotoxicity in microglia cells have not been well-examined. In this study, we examined the lipotoxic effects of PA on microglia cells, the potential role of FFAR1/FFAR4 and FA uptake in PA lipotoxicity, and how UFAs might protect microglia from PA lipotoxicity.

Our studies found that PA overload induced time- and dose-dependent decrease in microglia viability. PA also dose-dependently increased TUNEL^+^ cells, which suggests that these cells might be apoptotic. Considering that necrosis may also result in DNA strand breaks [50] and lead to TUNEL^+^ staining, we also stained cells with Annexin V-FITC and PI since phosphatidylserine exposure on the outside of cells is an early event of apoptosis. PI enters necrotic or late apoptotic cells but is excluded from early apoptotic cells, therefore Annexin V^+^/PI^−^ population represents early apoptotic cells [51]. In our studies, the population of Annexin V^+^/PI^−^ cells appeared to increase following PA treatment as compared to BSA controls, suggesting that PA overload may induce apoptosis. Annexin V^+^/PI^+^ population was found to be significantly greater than the Annexin V^+^/PI^−^ population. Considering that necrotic cells have ruptured membranes, which may allow Annexin V to access the internal plasma membrane leaflet and stain the cells, the Annexin V^+^/PI^+^ population may therefore also contain necrotic cells in addition to apoptotic cells. We also quantified the activity of lactate dehydrogenase (LDH) in the cell culture media. As shown in our Supplemental Figure S1, treatment with 200 µM PA for 24 h significantly increased LDH activity as compared to BSA-treated control, suggesting that PA lipotoxicity in microglia may also involve necrosis.

Both FFAR1 and FFAR2 have been suggested to act as receptors for long-chain fatty acids and play important roles in various physiological and pathological processes, including metabolic disorders [52]. While inhibition of FFAR1 by its antagonist, ANT203, protects human islets against chronic PA-induced decrease in insulin content and glucose-stimulated insulin secretion [53], a number of studies have suggested that PA’s lipotoxic effects may not be mediated by FFAR1 [54]. Some studies even reported lipoprotective effects from FFAR1 and FFAR4 activation [55,56]. Our studies found that FFAR1 and FFAR4 antagonists did not alleviate PA-induced decrease in cell viability, suggesting that PA lipotoxicity in microglia cells may not be mediated by FFARs.

Our studies found that PA lipotoxicity in microglia cells was dependent on fatty acid uptake. Treatment of microglia cells with SSO not only significantly decreased fatty acid uptake but also abolished PA-induced lipotoxicity, suggesting that PA lipotoxicity in microglia may be mediated by intracellular mechanisms rather than cell surface receptors. Each UFA tested, namely LA, OA, ALA, and DHA, was not by itself lipotoxic and was able to protect microglia cells against PA lipotoxicity. Consistently, LA, OA, and DHA significantly decreased fatty acid uptake in PA-treated cells. It is well-recognized that UFAs are susceptible to oxidation and that the extent of autoxidation correlates with the number of double bonds in UFAs [57]. Oxidized UFAs may exhibit anti-proliferative properties [58]. The lipid dialdehyde products of oxidized UFAs are substrates for aldose reductase (AR), and inhibition of AR activity reportedly protects BV2 cells from toxic models of oxidative stress [59]. Among the FFAs used in our studies, the number of double bonds in DHA, ALA, LA, OA, and PA is 6, 3, 2, 1, and 0, respectively. DHA, ALA, OA, and LA did not induce a marked decrease in cell viability at 24 h treatment of BV2 microglia cells. Instead, they all protected microglia against PA lipotoxicity, suggesting that UFA autoxidation may not have contributed to our observed effects.

We also found that UFAs induced a significant increase in the accumulation of neutral lipids and in the formation of LDs in microglia cells. Given their central role in lipid metabolism, LDs have been suggested to protect against various types of cellular stress, including lipotoxic stress [60]. Arachidonic acid (AA) has been suggested to protect C2C12 cells from PA-mediated lipotoxicity by channeling PA into neutral lipids in C2C12 [24]. However, Plotz et al. reported that the protective effects of UFAs against PA-induced lipotoxicity in rat insulin-producing cells are not dependent on UFA-induced LD formation [61]. Whether enhanced neutral lipid synthesis and LD formation contribute to UFA-mediated protection against PA lipotoxicity in microglia cells will need further investigation. Future studies on inhibiting or knocking down the activities of diacylglycerol acyltrans-ferases (DGAT1 and DGAT2), the enzymes catalyzing the synthesis of TAGs from DAGs [62], may help to shed light on whether neutral lipid synthesis is involved in UFA lipoprotection. It would also be interesting to examine whether LD formation is involved in UFA lipoprotection by knocking down the activities of perilipins and seipin, which reportedly play important roles in LD biogenesis [62].

Park et al. (2014) reported that PA treatment induces G_2_/M arrest, increases the production of reactive oxygen species (ROS), and enhances the levels of two endoplasmic reticulum (ER) stress-related proteins, namely C/EBP homologous protein (CHOP) and phosphorylated inositol requiring enzyme 1 alpha (pIRE1α), and that inhibition of ER stress substantially rescued PA-induced cytotoxicity and cell cycle defects in human Chang liver cells [63]. Other studies have also examined the mechanisms of PA lipotoxicity and how UFAs may protect against PA lipotoxicity. For example, Tumova et al. reported that the protective effects of UFAs on PA-induced toxicity are not mediated by peroxisome proliferator-activated receptor δ (PPARδ) activation in skeletal muscle cells [64]. UFAs may rescue PA lipotoxicity by effectively blocking PA-induced H_2_O_2_ formation in the peroxisomes of insulin-producing cells [65] and relieving PA-induced ER stress in HEPG2 cells [66]. In contrast, our studies found that PA treatment induced cell death without affecting the cell cycle profile (Supplemental Figure S2) and that treatment with 0.2, 1, or 5 mM N-acetylcysteine (NAC), an antioxidant, did not alleviate PA-induced lipotoxicity in BV2 microglia cells (Supplemental Figure S3), while 5 mM NAC was found to alleviate 400 µM PA-induced apoptosis in hepatocytes [67].

Furthermore, we found that 24 h treatment with 200 µM of any of the FFAs, including PA, LA, OA, ALA, and DHA, did not enhance the mRNA expression levels of ER stress sensors, such as CHOP and glucose regulatory protein 78 (GRP78), when compared with BSA-treated cells (Supplemental Figure S4). Consistently, inhibition of ER stress with 40 µM, 200 µM, and 1 mM 4-phenylbutyric acid (4-PBA) did not alleviate PA-induced lipotoxicity in BV2 cells (Supplemental Figure S4) while 4-PBA at 500 nM and 5 mM was able to attenuate PA-induced expression of CHOP and apoptosis in H9C2 cardiomyocytes [27,68], suggesting that PA may induce lipotoxicity via different pathways in BV2 cells. Osario et al. simulated an inflammatory environment for astrocytes using PA, and through this simulation, they found that PA may affect the flux rate of 586 reactions, including those involved in oxidative phosphorylation, histidine metabolism, and fatty acid degradation pathways in astrocytes when compared with the control astrocytes [69]. Considering the unique immune regulatory function of microglia cells, it would be interesting to examine how the metabolic pathways are affected in microglia cells and how PA-induced metabolic effects relate to their immune regulation and lipotoxicity.

In summary, our studies found that PA overload induced microglia lipotoxicity as indicated by decreased cell viability and increased cell death and that PA lipotoxicity in microglia cells was dependent on its uptake, suggesting that PA lipotoxicity may be mediated by intracellular mechanisms rather than cell surface receptors. Our studies also found that long-chain UFAs were not lipotoxic and were protective of microglia cells against PA lipotoxicity. This protective effect was associated with their inhibition of fatty acid uptake and increased synthesis and incorporation of fatty acids into neutral lipids in PA-treated cells as compared to BSA-treated cells. Considering the reports that obesity and metabolic syndrome are associated with cognitive decline and dementia [70,71,72] and the important role of microglial dystrophy in β-amyloid deposits due to futile microglial reaction to insoluble amyloid deposits [32], it is important to further study the mechanisms of PA lipotoxicity and the mechanisms of UFA lipoprotection in microglial cells.

## 4. Materials and Methods

### 4.1. Cell Culture

The murine microglia cell line BV2 was kindly provided by Dr. Jau-Shyong Hong from the National Institutes of Health. These cells were maintained in Dulbecco’s Modified Eagle’s Medium (DMEM; Corning, NY, USA), supplemented with 10% fetal calf serum (ThermoFisher Scientific; Waltham, MA, USA), and maintained at 37 °C in a humidified incubator with 5% CO_2_. All experiments were performed with cells passaged fewer than 20 times.

### 4.2. Preparation of Fatty Acids

The fatty acids (FAs), namely PA, LA, OA, ALA, and DHA, as well as FA-free, low endotoxin bovine serum albumin (BSA), were purchased from Sigma-Aldrich (St. Louis, MO, USA). First, each FA was dissolved in 100% ethanol at 400 mM, and BSA was dissolved in serum-free DMEM at 13.5% (*w*/*v*). An appropriate volume of 400 mM of each FA was added to 13.5% BSA to prepare a 5 mM FA stock solution with a molar ratio of 1:2.5 (FA: BSA) [73]. Considering that most of the FFA in serum is bound to albumin [74], the FAs were conjugated to BSA by sonication for 10 strokes and then mixing on a nutator for 3 h at room temperature. The resulting FA solution was then filtered through a 0.22 μm filter, aliquoted, and stored at −80 °C until use.

### 4.3. MTT Assay

BV2 cells were seeded in 96-well cell culture plates at 3000 cells/well and incubated overnight. Cells were then treated with 25, 50, 100, or 200 µM FAs or an equal volume of BSA in serum-free media at concentrations as indicated in the results section. At 2 h prior to the end of treatment, 10 μL of 5 mg/mL MTT (Sigma-Aldrich, St. Louis, MO, USA) was added to each well at a final concentration of 0.5 mg/mL and incubated for another 2 h to allow formation of sufficient formazan crystals. At the end of treatment, 100 μL of solubilization solution was then added to each well. Following complete solubilization of the purple formazan crystals, absorbance was read on the Varioskan LUX Multimode Microplate Reader (Thermofisher; Waltham, MA, USA) at 570 nm and 650 nm. The absorbance at 570 in each well was then subtracted by its absorbance at 650 nm to obtain the corrected absorbance values. The percent cell viability was calculated using the following formula: corrected absorbance of the treatment group/corrected absorbance of the BSA control X 100.

### 4.4. TUNEL Assay

Cells were seeded in 8-well chamber slides at 8000 cells/chamber. After overnight incubation, cells were treated with 25, 50, 100, or 200 µM PA or an equal volume of BSA control in serum-free media as indicated. After fixation with 4% paraformaldehyde for 30 m at room temperature, cells were rinsed, permeabilized with 0.2% Triton X-100 in PBS for 5 m, rinsed again, and then incubated with a reaction mixture containing terminal deoxynucleotidyl transferase (TdT) and fluorescein-12-dUTP (Roche; Indianapolis, IN, USA) for 60 m at 37 °C following manufacturer’s instructions. After rinsing, cells were stained with propidium iodide (PI) and visualized under FluoView FV1000 confocal microscope (Olympus; Center Valley, PA, USA).

### 4.5. Annexin V Assay

Annexin V assay was conducted following manufacturer’s instructions. Briefly, following treatments as indicated in the results section, cells were collected by trypsinization, spun down, and then washed with binding buffer (140 mM NaCl, 4 mM KCl, 0.75 mM MgCl_2_, 10 mM HEPES, 2.5 mM CaCl_2_) before being resuspended in 100 µL binding buffer with 5% Annexin V-FITC (BD Biosciences; San Jose, CA, USA) and 2 μg/mL propidium iodide (PI) and incubated at room temperature for 15 min in the dark. Then the cells were kept on ice, and the volume was adjusted to 500 µL followed by analysis on the MACSQuant Analyzer 10 flow cytometer (Miltenyi Biotec; Auburn, CA, USA). The fluorescent intensities of FITC and PI were quantified. The central population of forward scatter (FSC) vs. side scatter (SSC) flow cytometry events was inclusively gated to omit debris. Singlets were then sub-gated by FSC-A vs. FSC-H. Flow cytometry data were compensated and analyzed using FlowJo 10 (FlowJo).

### 4.6. Lactate Dehydrogenase (LDH) Assay

LDH is a stable cytosolic enzyme released upon cell lysis. Its activity in cell culture supernatants was analyzed using the CytoTox 96^®^ non-radioactive cytotoxicity kit (Promega; Madison, WI, USA) following manufacturer’s instructions. Briefly, at the end of treatment, cell culture supernatants were collected, and cells were lysed in 1X lysis solution to generate maximum LDH release. 50 μL of samples were mixed with 50 μL of the CytoTox 96^®^ Reagent, incubated in the dark for 30 min at room temperature. Then 50 μL of Stop Solution was added to stop the reaction, and the absorbance was measured at 490 nm on the Varioskan LUX Multimode Microplate Reader (Thermofisher; Waltham, MA, USA). The background absorbance of the culture media from wells without cells was then subtracted from experimental wells. Percent cytotoxicity was calculated using the following formula: net absorbance of experimental samples/net absorbance of maximum LDH release X 100.

### 4.7. Cell Cycle Analysis

Cells were seeded in 12-well plates at 1.0 × 10^5^ cells/well. After overnight incubation, cells were treated with 200 µM PA, LA, OA, ALA, DHA, or BSA in serum-free media for 24 h. At the end of treatment, cells were collected by trypsinization and washed with D-PBS prior to fixation with 70% ethanol on ice for at least 1 h. Ethanol-fixed cells were then spun down, washed, resuspended in D-PBS, and incubated with 1 μg/mL 4′,6-diamidino-2-phenylindole (DAPI) staining solution for 30 m in the dark on ice. DAPI fluorescence was then determined by flow cytometry on the MACSQuant Analyzer 10 flow cytometer (Miltenyi Biotec; Auburn, CA, USA). Flow cytometry events were gated, and data were analyzed using FlowJo10 (FlowJo; Ashland, OR, USA).

### 4.8. RNA Isolation

Total RNA from BV2 cells was isolated using the TRIzol reagent (Sigma-Aldrich; St. Louis, MO, USA) according to manufacturer’s instructions. The prepared RNA samples were dissolved in RNase-free water and stored at −80 °C.

### 4.9. Semi-Quantitative Reverse Transcriptase-Polymerase Chain Reaction (RT-PCR) Assay

RT-PCR was conducted as described before [75,76]. Briefly, cDNA was synthesized from 1 µg of total RNA using oligo (dT)_12–18_ primer and Moloney Murine Leukemia Virus (M-MLV) reverse transcriptase (Promega; Madison, WI, USA). After cDNA synthesis, PCR amplification was carried out using appropriate sense and antisense primers specific for mouse *GAPDH* (a house-keeping gene), *CHOP*, *GRP78*, *FFAR1*, and *FFAR4* synthesized by Eurofins Genomics (Huntville, AL) in a final volume of 20 µL containing 1 μL of cDNA, 1X PCR buffer, 0.2 μM of each sense and antisense primer, 0.2 mM of dNTPs, and 0.5 unit of Taq DNA polymerase (Applied Biosystems; Foster City, CA, USA) [75,76]. The forward (F) and reverse (R) primers for *FFAR1*, *FFAR4*, *CHOP*, *GRP78*, and *GAPDH* were 5′-TTGGTCATCACTGCCTTCTG-3′ (F) and 5′-CCCTGTGATGAGTCCCAACT-3′ (R); 5′-CTCTGAGAGCCACCAGATCC-3′ (F) and 5′-AAGAAAAGGGATGGCCAGAT-3′ (R); 5′-CTGGAAGCCTGGTATGAGGAT-3′ (F) and 5′-CAGGGTCAAGAGTAGTGAAGGT-3′ (R) [77]; 5′-AGTGGTGGCCACTAATGGAG-3′ (F) and 5′-CAATCCTTGCTTGATGCTGA-3′ (R) [77]; 5′-CGAACATCATCCCTGCATCCA-3′ (F) and 5′-CCCAGTGAGCTTCCCGTTCA-3′ (R), respectively. The reaction was heated to 94 °C for 5 min, followed by denaturation at 94 °C for 30 s, annealing at 57 °C for 30 s, and extension at 72 °C for 30 s for 35 cycles, 30 cycles, 29 cycles, 28 cycles, and 24 cycles, respectively, for *FFAR1*, *FFAR4*, *CHOP*, *GRP78*, and *GAPDH*. After the final cycle, a 7 min extension step at 72 °C was included. PCR products were then run on a 2.0% agarose gel, and the gel image was recorded using the FluorChem system (Protein Sample; San Jose, CA, USA). The band intensities of genes of interest were digitized using VisionWorks^TM^ LS software (UVP; Upland, CA, USA) and normalized against the intensity of *GAPDH* in the same sample.

### 4.10. Analysis of the Effects of FFA Overload on Fatty Acid Uptake by Flow Cytometry

To examine the effects of FFA overload on FA uptake using flow cytometry, cells were seeded in 48-well cell culture plates at 0.25 × 10^5^ cells/well. Following overnight incubation, cells were treated with 200 µM FAs or BSA for 6 h in serum-free media as indicated in the results section and then incubated with 15 μM 4, 4-difluoro-5,7-dimethyl-4-bora-3a,4a-diaza-*s*-indacene-3-dodecanoic acid (BODIPY^TM^ FL C12) (ThermoFisher Scientific; Waltham, MA, USA), a fluorescent long-chain saturated fatty acid analogue, in serum-free media for 30 min at 37 °C. BODIPY^TM^ FL C12 was deemed to resemble the length of C16 FA since the BODIPY^TM^ FL fluorophore contributes an additional four-carbon length of acyl chain while BODIPY^TM^ FL C16 would be equivalent to the C20:0 arachidic acid. Furthermore, metabolic tracing performed in zebrafish has revealed that the complex lipid product profile of ^3^H-PA most closely resembles that of BODIPY^TM^ FL C12, rather than BODIPY^TM^ FL C16 [40]. Therefore, BODIPY^TM^ FL C12 was deemed as a suitable fluorescent PA analogue. Considering that PA is the most abundant FA in plasma [42], BODIPY^TM^ FL C12 was used in our FA uptake assay. At the end of treatment, cells were collected by trypsinization, rinsed with ice-cold D-PBS, spun down, resuspended in 200 µL ice-cold D-PBS, and immediately analyzed by flow cytometry.

### 4.11. Analysis of the Effects of FFAs on Neutral Lipid Contents by Flow Cytometry

To analyze microglial neutral lipid content by flow cytometry, cells were seeded in 24-well plates at a density of 0.5 × 10^5^ cells/well. After overnight incubation, cells were treated with 200 µM FFAs or BSA in serum-free media for 6 h. Following treatment, cells were incubated with 5 μM 4,4-difluoro-1,3,5,7,8-pentamethyl-4-bora-3a,4a-diaza-*s*-indacene (BODIPY^TM^ 493/503; ThermoFisher; Waltham, MA, USA), a fluorescent marker for neutral lipids, in serum-free media for 30 min at 37 °C. Cells were rinsed with PBS, then trypsinized, rinsed with PBS again, resuspended in 400 µL ice-cold PBS, and analyzed by flow cytometry.

### 4.12. Visualization of Fatty Acid Uptake and Neutral Lipid Accumulation Using Confocal Microscopy

To visualize the localization of BODIPY^TM^ FL C12 and its incorporation into neutral lipids in microglia treated with FFAs, microglia cells were seeded on glass coverslips in 24-well plates at 2.5 × 10^4^ cells/well, incubated overnight, and treated with 200 µM FFAs or BSA in serum-free media for 6 h. Following treatment, cells were incubated with 15 μM BODIPY^TM^ FL C12 (ThermoFisher Scientific; Waltham, MA, USA) and 2 μM Nile Red (Cayman Chemical; Ann Arbor, MI, USA) in serum-free media for 30 min at 37 °C. Cells were then rinsed with D-PBS, fixed with 4% paraformaldehyde for 20 min at 4 °C, rinsed with D-PBS again, and stained with 1 μg/mL 4′,6-diamidino-2-phenylindole (DAPI) in D-PBS for 5 min. Cells were then rinsed with D-PBS again, and then the coverslips were mounted onto glass slides and visualized under confocal microscopy.

### 4.13. Statistical Analysis

Data were analyzed using GraphPad Prism 6 (GraphPad Software; San Diego, CA, USA). *p* < 0.05 was considered statistically significant.

## Data Availability

The data presented in this study are available in this article and its supplementary material.

## Supplementary Materials

The following are available online at https://www.mdpi.com/article/10.3390/ijms22169093/s1, Figure S1: LDH cytotoxicity assay of BV2 cells following treatment with BSA and PA, Figure S2: Cell cycle analysis of BV2 cells following treatment with different FFAs, Figure S3: Viability of BV2 cells pre-treated with NAC followed by treatment with BSA or PA, Figure S4: Relative mRNA expression of CHOP and GRP78 in BV2 cells following 24 h treatment with different FFAs and viability of BV2 cells pre-treated with 4-PBA followed by treatment with BSA or PA for 24 h.

## References

[1] Hussain G., Schmitt F., Loeffler J.P., de Aguilar J.L. (2013). Fatting the brain: A brief of recent research. Front. Cell. Neurosci..

[2] Engin A.B. (2017). What Is Lipotoxicity?. Adv. Exp. Med. Biol..

[3] Karmi A., Iozzo P., Viljanen A., Hirvonen J., Fielding B.A., Virtanen K., Oikonen V., Kemppainen J., Viljanen T., Guiducci L. (2010). Increased brain fatty acid uptake in metabolic syndrome. Diabetes.

[4] Quehenberger O., Armando A.M., Brown A.H., Milne S.B., Myers D.S., Merrill A.H., Bandyopadhyay S., Jones K.N., Kelly S., Shaner R.L. (2010). Lipidomics reveals a remarkable diversity of lipids in human plasma. J. Lipid Res..

[5] Carta G., Murru E., Lisai S., Sirigu A., Piras A., Collu M., Batetta B., Gambelli L., Banni S. (2015). Dietary triacylglycerols with palmitic acid in the sn-2 position modulate levels of N-acylethanolamides in rat tissues. PLoS ONE.

[6] Melo H.M., Seixas da Silva G.D.S., Sant’Ana M.R., Teixeira C.V.L., Clarke J.R., Miya Coreixas V.S., de Melo B.C., Fortuna J.T.S., Forny-Germano L., Ledo J.H. (2020). Palmitate Is Increased in the Cerebrospinal Fluid of Humans with Obesity and Induces Memory Impairment in Mice via Pro-inflammatory TNF-alpha. Cell Rep..

[7] Dragano N.R., Monfort-Pires M., Velloso L.A. (2020). Mechanisms Mediating the Actions of Fatty Acids in the Hypothalamus. Neuroscience.

[8] Firlag M., Kamaszewski M., Gaca K., Adamek D., Balasinska B. (2013). The neuroprotective effect of long-term n-3 polyunsaturated fatty acids supplementation in the cerebral cortex and hippocampus of aging rats. Folia Neuropathol..

[9] Jia D., Heng L.J., Yang R.H., Gao G.D. (2014). Fish oil improves learning impairments of diabetic rats by blocking PI3K/AKT/nuclear factor-kappaB-mediated inflammatory pathways. Neuroscience.

[10] Mancini A.D., Poitout V. (2013). The fatty acid receptor FFA1/GPR40 a decade later: How much do we know?. Trends Endocrinol. Metab. TEM.

[11] Zhou Y.J., Song Y.L., Zhou H., Li Y. (2012). Linoleic acid activates GPR40/FFA1 and phospholipase C to increase [Ca2+]i release and insulin secretion in islet beta-cells. Chin. Med. Sci. J..

[12] Kim J.Y., Lee H.J., Lee S.J., Jung Y.H., Yoo D.Y., Hwang I.K., Seong J.K., Ryu J.M., Han H.J. (2017). Palmitic Acid-BSA enhances Amyloid-beta production through GPR40-mediated dual pathways in neuronal cells: Involvement of the Akt/mTOR/HIF-1alpha and Akt/NF-kappaB pathways. Sci. Rep..

[13] Milligan G., Alvarez-Curto E., Hudson B.D., Prihandoko R., Tobin A.B. (2017). FFA4/GPR120: Pharmacology and Therapeutic Opportunities. Trends Pharmacol. Sci..

[14] Kimura I., Ichimura A., Ohue-Kitano R., Igarashi M. (2020). Free Fatty Acid Receptors in Health and Disease. Physiol. Rev..

[15] Ohue-Kitano R., Yasuoka Y., Goto T., Kitamura N., Park S.B., Kishino S., Kimura I., Kasubuchi M., Takahashi H., Li Y. (2018). alpha-Linolenic acid-derived metabolites from gut lactic acid bacteria induce differentiation of anti-inflammatory M2 macrophages through G protein-coupled receptor 40. FASEB J. Off. Publ. Fed. Am. Soc. Exp. Biol..

[16] Sparks S.M., Chen G., Collins J.L., Danger D., Dock S.T., Jayawickreme C., Jenkinson S., Laudeman C., Leesnitzer M.A., Liang X. (2014). Identification of diarylsulfonamides as agonists of the free fatty acid receptor 4 (FFA4/GPR120). Bioorganic Med. Chem. Lett..

[17] Wu J., Sun P., Zhang X., Liu H., Jiang H., Zhu W., Wang H. (2012). Inhibition of GPR40 protects MIN6 beta cells from palmitate-induced ER stress and apoptosis. J. Cell. Biochem..

[18] Wang Y., Xie T., Zhang D., Leung P.S. (2019). GPR120 protects lipotoxicity-induced pancreatic beta-cell dysfunction through regulation of PDX1 expression and inhibition of islet inflammation. Clin. Sci..

[19] Dragano N.R.V., Solon C., Ramalho A.F., de Moura R.F., Razolli D.S., Christiansen E., Azevedo C., Ulven T., Velloso L.A. (2017). Polyunsaturated fatty acid receptors, GPR40 and GPR120, are expressed in the hypothalamus and control energy homeostasis and inflammation. J. Neuroinflamm..

[20] Mo Z., Tang C., Li H., Lei J., Zhu L., Kou L., Luo S., Li C., Chen W., Zhang L. (2020). Eicosapentaenoic acid prevents inflammation induced by acute cerebral infarction through inhibition of NLRP3 inflammasome activation. Life Sci..

[21] Low Y.L., Pan Y., Short J.L., Nicolazzo J.A. (2021). Profiling the expression of fatty acid-binding proteins and fatty acid transporters in mouse microglia and assessing their role in docosahexaenoic acid-d5 uptake. Prostaglandins Leukot. Essent. Fat. Acids.

[22] Guo Y., Cordes K.R., Farese R.V., Walther T.C. (2009). Lipid droplets at a glance. J. Cell Sci..

[23] Welte M.A., Gould A.P. (2017). Lipid droplet functions beyond energy storage. Biochim. Et Biophys. Acta Mol. Cell Biol. Lipids.

[24] Cheon H.G., Cho Y.S. (2014). Protection of palmitic acid-mediated lipotoxicity by arachidonic acid via channeling of palmitic acid into triglycerides in C2C12. J. Biomed. Sci..

[25] Almaguel F.G., Liu J.W., Pacheco F.J., Casiano C.A., De Leon M. (2009). Activation and reversal of lipotoxicity in PC12 and rat cortical cells following exposure to palmitic acid. J. Neurosci. Res..

[26] Li P., Li L., Zhang C., Cheng X., Zhang Y., Guo Y., Long M., Yang S., He J. (2019). Palmitic Acid and beta-Hydroxybutyrate Induce Inflammatory Responses in Bovine Endometrial Cells by Activating Oxidative Stress-Mediated NF-kappaB Signaling. Molecules.

[27] Yang L., Guan G., Lei L., Liu J., Cao L., Wang X. (2019). Oxidative and endoplasmic reticulum stresses are involved in palmitic acid-induced H9c2 cell apoptosis. Biosci. Rep..

[28] Yuzefovych L., Wilson G., Rachek L. (2010). Different effects of oleate vs. palmitate on mitochondrial function, apoptosis, and insulin signaling in L6 skeletal muscle cells: Role of oxidative stress. Am. J. Physiol. Endocrinol. Metab..

[29] Suzuki E., Matsuda T., Kawamoto T., Takahashi H., Mieda Y., Matsuura Y., Takai T., Kanno A., Koyanagi-Kimura M., Asahara S.I. (2018). Docosahexaenoic Acid Reduces Palmitic Acid-Induced Endoplasmic Reticulum Stress in Pancreatic Beta Cells. Kobe J. Med Sci..

[30] Leyrolle Q., Laye S., Nadjar A. (2019). Direct and indirect effects of lipids on microglia function. Neurosci. Lett..

[31] Lawson L.J., Perry V.H., Dri P., Gordon S. (1990). Heterogeneity in the distribution and morphology of microglia in the normal adult mouse brain. Neuroscience.

[32] Streit W.J., Khoshbouei H., Bechmann I. (2020). Dystrophic microglia in late-onset Alzheimer’s disease. Glia.

[33] Tracy L.M., Bergqvist F., Ivanova E.V., Jacobsen K.T., Iverfeldt K. (2013). Exposure to the saturated free fatty acid palmitate alters BV-2 microglia inflammatory response. J. Mol. Neurosci. MN.

[34] Beaulieu J., Costa G., Renaud J., Moitie A., Glemet H., Sergi D., Martinoli M.G. (2021). The Neuroinflammatory and Neurotoxic Potential of Palmitic Acid Is Mitigated by Oleic Acid in Microglial Cells and Microglial-Neuronal Co-cultures. Mol. Neurobiol..

[35] Chausse B., Kakimoto P.A., Caldeira-da-Silva C.C., Chaves-Filho A.B., Yoshinaga M.Y., da Silva R.P., Miyamoto S., Kowaltowski A.J. (2019). Distinct metabolic patterns during microglial remodeling by oleate and palmitate. Biosci. Rep..

[36] Briscoe C.P., Tadayyon M., Andrews J.L., Benson W.G., Chambers J.K., Eilert M.M., Ellis C., Elshourbagy N.A., Goetz A.S., Minnick D.T. (2003). The orphan G protein-coupled receptor GPR40 is activated by medium and long chain fatty acids. J. Biol. Chem..

[37] Oh D.Y., Lagakos W.S. (2011). The role of G-protein-coupled receptors in mediating the effect of fatty acids on inflammation and insulin sensitivity. Curr. Opin. Clin. Nutr. Metab. Care.

[38] Talukdar S., Olefsky J.M., Osborn O. (2011). Targeting GPR120 and other fatty acid-sensing GPCRs ameliorates insulin resistance and inflammatory diseases. Trends Pharmacol. Sci..

[39] Kuda O., Pietka T.A., Demianova Z., Kudova E., Cvacka J., Kopecky J., Abumrad N.A. (2013). Sulfo-N-succinimidyl oleate (SSO) inhibits fatty acid uptake and signaling for intracellular calcium via binding CD36 lysine 164: SSO also inhibits oxidized low density lipoprotein uptake by macrophages. J. Biol. Chem..

[40] Quinlivan V.H., Wilson M.H., Ruzicka J., Farber S.A. (2017). An HPLC-CAD/fluorescence lipidomics platform using fluorescent fatty acids as metabolic tracers. J. Lipid Res..

[41] Malhi H., Gores G.J. (2008). Molecular mechanisms of lipotoxicity in nonalcoholic fatty liver disease. Semin. Liver Dis..

[42] Abdelmagid S.A., Clarke S.E., Nielsen D.E., Badawi A., El-Sohemy A., Mutch D.M., Ma D.W. (2015). Comprehensive profiling of plasma fatty acid concentrations in young healthy Canadian adults. PLoS ONE.

[43] de Vries J.E., Vork M.M., Roemen T.H., de Jong Y.F., Cleutjens J.P., van der Vusse G.J., van Bilsen M. (1997). Saturated but not mono-unsaturated fatty acids induce apoptotic cell death in neonatal rat ventricular myocytes. J. Lipid Res..

[44] Cnop M., Hannaert J.C., Hoorens A., Eizirik D.L., Pipeleers D.G. (2001). Inverse relationship between cytotoxicity of free fatty acids in pancreatic islet cells and cellular triglyceride accumulation. Diabetes.

[45] Wei Y., Wang D., Topczewski F., Pagliassotti M.J. (2006). Saturated fatty acids induce endoplasmic reticulum stress and apoptosis independently of ceramide in liver cells. Am. J. Physiol. Endocrinol. Metab..

[46] Cao J., Dai D.L., Yao L., Yu H.H., Ning B., Zhang Q., Chen J., Cheng W.H., Shen W., Yang Z.X. (2012). Saturated fatty acid induction of endoplasmic reticulum stress and apoptosis in human liver cells via the PERK/ATF4/CHOP signaling pathway. Mol. Cell. Biochem..

[47] Sieber J., Lindenmeyer M.T., Kampe K., Campbell K.N., Cohen C.D., Hopfer H., Mundel P., Jehle A.W. (2010). Regulation of podocyte survival and endoplasmic reticulum stress by fatty acids. Am. J. Physiol. Renal. Physiol..

[48] Mayer C.M., Belsham D.D. (2010). Palmitate attenuates insulin signaling and induces endoplasmic reticulum stress and apoptosis in hypothalamic neurons: Rescue of resistance and apoptosis through adenosine 5’ monophosphate-activated protein kinase activation. Endocrinology.

[49] Zhou H., Urso C.J., Jadeja V. (2020). Saturated Fatty Acids in Obesity-Associated Inflammation. J. Inflamm. Res..

[50] Labat-Moleur F., Guillermet C., Lorimier P., Robert C., Lantuejoul S., Brambilla E., Negoescu A. (1998). TUNEL apoptotic cell detection in tissue sections: Critical evaluation and improvement. J. Histochem. Cytochem. Off. J. Histochem. Soc..

[51] Crowley L.C., Marfell B.J., Scott A.P., Waterhouse N.J. (2016). Quantitation of Apoptosis and Necrosis by Annexin V Binding, Propidium Iodide Uptake, and Flow Cytometry. Cold Spring Harb. Protoc..

[52] Hara T., Hirasawa A., Ichimura A., Kimura I., Tsujimoto G. (2011). Free fatty acid receptors FFAR1 and GPR120 as novel therapeutic targets for metabolic disorders. J. Pharm. Sci..

[53] Kristinsson H., Smith D.M., Bergsten P., Sargsyan E. (2013). FFAR1 is involved in both the acute and chronic effects of palmitate on insulin secretion. Endocrinology.

[54] Lan H., Hoos L.M., Liu L., Tetzloff G., Hu W., Abbondanzo S.J., Vassileva G., Gustafson E.L., Hedrick J.A., Davis H.R. (2008). Lack of FFAR1/GPR40 does not protect mice from high-fat diet-induced metabolic disease. Diabetes.

[55] Panse M., Gerst F., Kaiser G., Teutsch C.A., Dolker R., Wagner R., Haring H.U., Ullrich S. (2015). Activation of extracellular signal-regulated protein kinases 1 and 2 (ERK1/2) by free fatty acid receptor 1 (FFAR1/GPR40) protects from palmitate-induced beta cell death, but plays no role in insulin secretion. Cell. Physiol. Biochem. Int. J. Exp. Cell. Physiol. Biochem. Pharmacol..

[56] Christiansen E., Watterson K.R., Stocker C.J., Sokol E., Jenkins L., Simon K., Grundmann M., Petersen R.K., Wargent E.T., Hudson B.D. (2015). Activity of dietary fatty acids on FFA1 and FFA4 and characterisation of pinolenic acid as a dual FFA1/FFA4 agonist with potential effect against metabolic diseases. Br. J. Nutr..

[57] Cosgrove J.P., Church D.F., Pryor W.A. (1987). The kinetics of the autoxidation of polyunsaturated fatty acids. Lipids.

[58] Iuchi K., Ema M., Suzuki M., Yokoyama C., Hisatomi H. (2019). Oxidized unsaturated fatty acids induce apoptotic cell death in cultured cells. Mol. Med. Rep..

[59] Elmazoglu Z., Prnova M.S., Stefek M., Ceylan A.F., Aschner M., Rangel-Lopez E., Santamaria A., Karasu C. (2021). Protective Effects of Novel Substituted Triazinoindole Inhibitors of Aldose Reductase and Epalrestat in Neuron-like PC12 Cells and BV2 Rodent Microglial Cells Exposed to Toxic Models of Oxidative Stress: Comparison with the Pyridoindole Antioxidant Stobadine. Neurotox. Res..

[60] Jarc E., Petan T. (2019). Lipid Droplets and the Management of Cellular Stress. Yale J. Biol. Med..

[61] Plotz T., Hartmann M., Lenzen S., Elsner M. (2016). The role of lipid droplet formation in the protection of unsaturated fatty acids against palmitic acid induced lipotoxicity to rat insulin-producing cells. Nutr. Metab..

[62] Olzmann J.A., Carvalho P. (2019). Dynamics and functions of lipid droplets. Nat. Rev. Mol. Cell Biol..

[63] Park E.J., Lee A.Y., Park S., Kim J.H., Cho M.H. (2014). Multiple pathways are involved in palmitic acid-induced toxicity. Food Chem. Toxicol. Int. J. Publ. Br. Ind. Biol. Res. Assoc..

[64] Tumova J., Malisova L., Andel M., Trnka J. (2015). Protective Effect of Unsaturated Fatty Acids on Palmitic Acid-Induced Toxicity in Skeletal Muscle Cells is not Mediated by PPARdelta Activation. Lipids.

[65] Gehrmann W., Wurdemann W., Plotz T., Jorns A., Lenzen S., Elsner M. (2015). Antagonism Between Saturated and Unsaturated Fatty Acids in ROS Mediated Lipotoxicity in Rat Insulin-Producing Cells. Cell. Physiol. Biochem. Int. J. Exp. Cell. Physiol. Biochem. Pharmacol..

[66] Zeng X., Zhu M., Liu X., Chen X., Yuan Y., Li L., Liu J., Lu Y., Cheng J., Chen Y. (2020). Oleic acid ameliorates palmitic acid induced hepatocellular lipotoxicity by inhibition of ER stress and pyroptosis. Nutr. Metab..

[67] Egnatchik R.A., Leamy A.K., Noguchi Y., Shiota M., Young J.D. (2014). Palmitate-induced activation of mitochondrial metabolism promotes oxidative stress and apoptosis in H4IIEC3 rat hepatocytes. Metab. Clin. Exp..

[68] Zou L., Li X., Wu N., Jia P., Liu C., Jia D. (2017). Palmitate induces myocardial lipotoxic injury via the endoplasmic reticulum stressmediated apoptosis pathway. Mol. Med. Rep..

[69] Osorio D., Pinzon A., Martin-Jimenez C., Barreto G.E., Gonzalez J. (2019). Multiple Pathways Involved in Palmitic Acid-Induced Toxicity: A System Biology Approach. Front. Neurosci..

[70] Dye L., Boyle N.B., Champ C., Lawton C. (2017). The relationship between obesity and cognitive health and decline. Proc. Nutr. Soc..

[71] Ivanova N., Liu Q., Agca C., Agca Y., Noble E.G., Whitehead S.N., Cechetto D.F. (2020). White matter inflammation and cognitive function in a co-morbid metabolic syndrome and prodromal Alzheimer’s disease rat model. J. Neuroinflamm..

[72] Lee J.E., Shin D.W., Han K., Kim D., Yoo J.E., Lee J., Kim S., Son K.Y., Cho B., Kim M.J. (2020). Changes in Metabolic Syndrome Status and Risk of Dementia. J. Clin. Med..

[73] Urso C.J.Z.H. (2021). Differential Effects of Unsaturated Fatty Acids and Saturated Fatty Acids in Lipotoxicity and Neutral Lipid Accumulation in Neuro-2a Cells. Biomed. J. Sci. Tech. Res..

[74] Richieri G.V., Kleinfeld A.M. (1995). Unbound free fatty acid levels in human serum. J. Lipid Res..

[75] Le Rouzic V., Corona J., Zhou H. (2011). Postnatal development of hepatic innate immune response. Inflammation.

[76] Ortega A., Jadeja V., Zhou H. (2011). Postnatal development of lipopolysaccharide-induced inflammatory response in the brain. Inflamm. Res..

[77] Barabas P., Liu A., Xing W., Chen C.K., Tong Z., Watt C.B., Jones B.W., Bernstein P.S., Krizaj D. (2013). Role of ELOVL4 and very long-chain polyunsaturated fatty acids in mouse models of Stargardt type 3 retinal degeneration. Proc. Natl. Acad. Sci. USA.

