# Supplementary material for: Palmitic Acid Lipotoxicity in Microglia Cells Is Ameliorated by Unsaturated Fatty Acids
Source: Int J Mol Sci. 2021 Aug 23;22(16):9093. doi: 10.3390/ijms22169093 (PMC8396597; doi:10.3390/ijms22169093)
Supplement: Supplementary file 1 [file ijms-22-09093-s001.zip › ijms-1292719-supplementary.pdf]

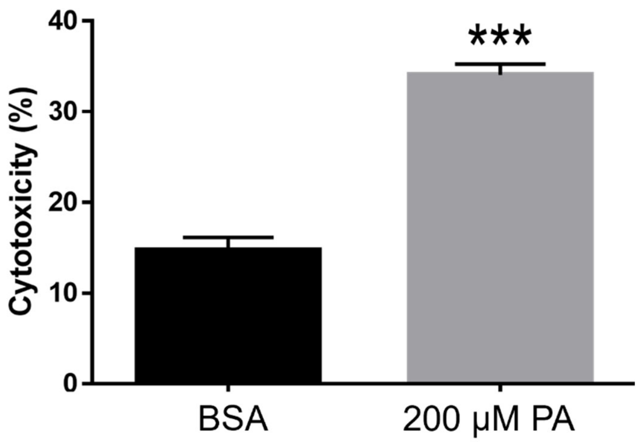

Supplemental Figure S1. LDH cytotoxicity assay of BV2 cells following treatment with BSA and PA. BV2 cells were treated with BSA or 200 μM PA in serum free media for 24 h, and percent cytotoxicity was analyzed based on LDH activity. The values presented were representative of three independent experiments with triplicate measurements (mean ± SD). Statistical analysis was performed using unpaired t-test. *** $p < 0.001$ vs. BSA control.

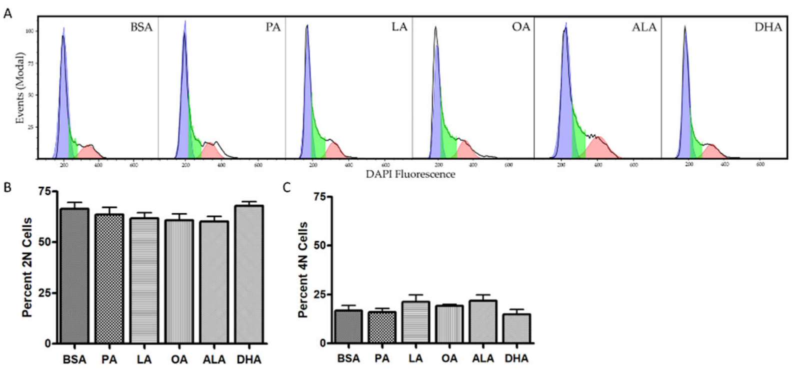

Supplemental Figure S2. Cell cycle analysis of BV2 cells following treatment with different FFAs. BV2 cells were treated with 200 μM PA, LA, OA, ALA, DHA, or equal volume of BSA control in serum free media for 24 h and cell cycle profile was analyzed by DAPI staining via flow cytometry with histograms shown in A and quantitated percentage of 2N and 4N cells shown in B and C respectively.

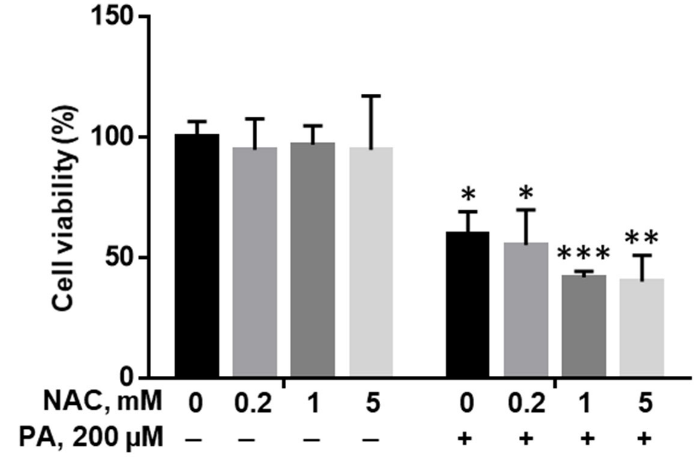

Supplemental Figure S3. Viability of BV2 cells pre-treated with NAC followed by treatment with BSA or PA. BV2 cells were pre-treated with 0, 0.2, 1, or 5 mM NAC, an antioxidant, for 1 h followed by treatment with 200 μM PA or equivalent volume of BSA in serum free media for 24 h, and their viability was assessed by MTT assay. Data were analyzed using two-way ANOVA followed by Tukey's multiple comparison test. The values presented were representative of three independent experiments with triplicate measurements (mean ± SD). * $p < 0.05$ vs. corresponding BSA control; ** $p < 0.01$ vs. corresponding BSA control; *** $p < 0.001$ vs. corresponding BSA control.

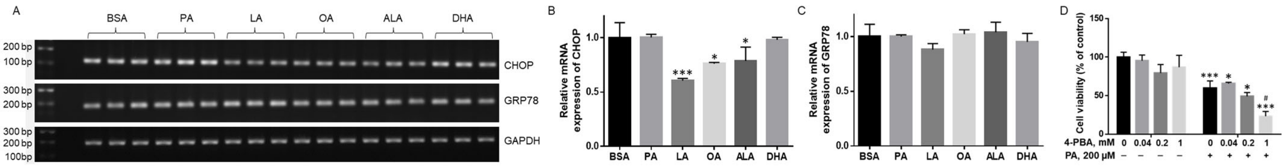

Supplemental Figure S4. Relative mRNA expression of CHOP and GRP78 in BV2 cells following 24 h treatment with different FFAs and viability of BV2 cells pre-treated with 4-PBA followed by treatment with BSA or PA for 24 h. (A-C) BV2 cells were treated with 200 μM PA, LA, OA, ALA, DHA or equal volume of BSA control in serum free media for 24 h, and their relative mRNA levels of CHOP and GRP78 was measured by semi-quantitative RT-PCR. Data were analyzed using one-way ANOVA followed by Dunnett's multiple comparison test. (D) BV2 cells were pre-treated with different concentrations of 4-PBA for 1 h followed by treatment with 200 μM PA or equal volume of BSA control in serum free media for 24 h, and their viability was assessed by MTT assay. Data were analyzed using two-way ANOVA followed by Tukey's multiple comparison test. The values presented were representative of three independent experiments with triplicate measurements (mean ± SD). *** $p < 0.001$ vs. BSA control; * $p < 0.05$ vs. BSA control; # $p < 0.05$ vs. 200 μM PA.
